# Early coronary angiography in patients with myocardial infarction without ST elevation after out-of-hospital cardiac arrest: a systematic review and meta-analysis

**DOI:** 10.3389/fcvm.2024.1374619

**Published:** 2024-11-20

**Authors:** Warda Ahmed, Arooba Ejaz, Muhammad Sameer Arshad, Manahil Mubeen, Aymen Ahmed, Asad Ali Siddiqui, Zoaib Habib Tharwani, F. N. U. Deepak, Prince Kumar, Izza Shahid, Muhammad Mustafa Memon

**Affiliations:** ^1^ Department of Medicine, Aga Khan University, Karachi, Pakistan; ^2^ Dow Medical College, Dow University of Health Sciences, Karachi, Pakistan; ^3^ Department of Medicine, Benazir Bhutto Shaheed University Lyari, Karachi, Pakistan; ^4^ Department of Cardiovascular Sciences, Houston Methodist Hospital, Houston, OH, United States; ^5^ Department of Medicine, Rochester General Hospital, Rochester, MI, United States

**Keywords:** OHCA, coronary angiography, myocardial infarction, NSTE, CAD

## Abstract

**Background:**

Early coronary angiography (CAG) in post-cardiac arrest patients without ST-segment elevation is a topic of debate. This meta-analysis aimed to assess its impact on outcomes.

**Methods:**

A search of Medline and Cochrane up to February 2023 was conducted to identify randomized controlled trials and observational studies comparing patients undergoing early CAG vs. delayed/no CAG after experiencing out-of-hospital cardiac arrest. A random-effects model pooled odds ratios (ORs) with 95% confidence intervals (CIs). Meta-regression explored factors modifying effect sizes.

**Results:**

We identified 16 studies (7 RCTs, 9 observational studies) involving 4,737 patients. Early CAG significantly reduced long-term mortality [OR: 0.66 (0.51–0.85)], and increased favorable cerebral performance category (CPC) 1–2 at discharge [OR: 1.49 (1.09–2.03)]. Observational study subgroup showed decreased short-term mortality, long-term mortality, and CPC 1–2 at discharge, unlike RCT subgroup. Meta-regression revealed type 2 diabetes mellitus and follow-up time influencing short-term mortality and CPC 1–2 at discharge, respectively.

**Conclusion:**

Early CAG in post-cardiac arrest patients without ST elevation is associated with long-term clinical benefits, particularly evident in observational studies. Interpretation should be cautious.

## Introduction

Out of hospital cardiac arrest (OHCA) annually affects around 3.8 million people worldwide with as many as a thousand suffering from OHCA every day in the United States alone (1, 2). Despite progressive advances in resuscitation care, survival rates stand at only 8%–12% in OHCA patients (3).

Significant coronary artery disease (CAD) (at least 1 stenosis >70%) is prevalent in 70%–95% of OHCA patients with an initially shockable rhythm and evidence of ST segment elevation (STE) on post resuscitation ECG (4). In contrast, significant CAD is only found in 25%–60% of OHCA patients with a shockable rhythm but no STE (NSTE) on post resuscitation ECG. This implies that in patients with NSTE, a coronary lesion may not be the only reason for cardiac arrest. Therefore, both the American Heart Association (AHA) and European Society of Cardiology (ESC) pertinently recommend early coronary artery angiography (CAG) in OHCA patients with STE since it would help in detecting a coronary artery lesion, the factor precipitating cardiac arrest (5, 6) However, in the same context, performing an emergency CAG in patients with NSTE is less likely to detect CAD and may only serve to unnecessarily delay critical ascertainment of the actual diagnosis and emergency management.

Current guidelines generally remain silent about the role of emergency CAG in OHCA patients with NSTE. However, they do specify a subset of these patients who should be indicated for an early CAG. The ESC guidelines recommend emergency CAG in highly suspicious patients while the AHA guidelines consider early CAG advisable in metabolically or hemodynamically unstable comatose patients with NSTE (5, 6). Meanwhile, data from studies conducted in OHCA patients with NSTE conflict over whether early CAG may help improve patient outcomes. The most recent meta-analysis of randomized controlled trials (RCTs) depicted no significant prognostic differences between early and delayed CAG (7). However, notably, it did not differentiate between short and long-term mortality and furthermore, analysed the composite outcome of all-cause death and neurological deficit, thereby not providing a directly accurate idea of how neurological functional recovery compares between early and delayed CAG NSTE OHCA patients.

Therefore, given the heterogenous findings and paucity of evidence, we conducted a meta-analysis of both randomized control trials (RCTs) and observational studies to compare patient outcomes on performing early CAG vs. delayed/no CAG after OHCA in patients with NSTE on post resuscitation ECG. Additionally, baseline comorbidities may likely influence patient outcomes after CAG and hence, to enable a more holistic comparison, we also analysed the interaction of outcomes in the two arms with pertinent baseline comorbidities such as type 2 diabetes mellitus (T2DM) and hypertension.

## Methods

This study was conducted while adhering to the Preferred Reporting Items for Systematic Reviews and Meta-Analyses (PRISMA) and Cochrane guidelines (8, 9).

### Search strategy

The MEDLINE and Cochrane databases were comprehensively searched in the last week of February 2023 to identify all articles since inception of the database. No time or language barriers were set, and the detailed search strategy is mentioned in Supplementary Table 1.

### Study selection criteria

The inclusion criteria consisted of (1) Studies that were RCTs or observational studies; (2) Studies comprising of OHCA patients; (3) Studies comparing patients undergoing early CAG to those undergoing delayed or no CAG; and (4) Studies reporting at least one of our outcomes of interest.

The exclusion criteria consisted of (1) Studies without an adequate control arm; (2) Studies consisting of patients without OHCA; (3) Studies not reporting any of our outcomes of interest.

### Screening process and data extraction

Two reviewers (W.A and A.E) independently screened the articles based on their titles and abstracts, followed by which the full text was read and reviewed to ascertain relevance. Any conflicts were resolved by a third reviewer (M.S.A). Subsequently, W.A and A.E independently extracted the data from the shortlisted articles, with any discrepancies being resolved by a third reviewer (M.S.A).

### Outcomes of interest

The primary outcomes of interest were short-term and long-term mortality. Short-term mortality was defined as mortality until hospital discharge, while long-term mortality referred to the cumulative deaths from time of hospital discharge till the longest available follow-up. Secondary outcomes were cerebral performance category (CPC) 1–2 at discharge, CPC 1–2 at the longest available follow-up, and the occurrence of percutaneous coronary intervention after CAG. Additionally, baseline demographics of the included studies were extracted and are detailed in Table 1.

**Table 1:** Baseline characteristics of included studies.

Study name and year	Study design	Group	Timing of CAG (early, delayed)	No. of participants in each group	Age (Years)	Sex Men (%)	BLS (%)	VF/VT (%)	PCI (%)	GCS
Bro-Jeppesen et al. (2012)	Observational	Early/late or No CAG	≤12 h, 12–30 days	82/162	59/62	82/78	57/56	90/68	29/15	≤8
Dankiewicz et al. (2015)	Observational	Early/late or No CAG	Not available	252/292	65/68	81/78	73/68	80/71	40/9	N/A
Garcia et al. (2015)	Observational	Early/late or No CAG	<6 h, not specified	231/84	56/54	77/77	N/A	100/100	12/12	N/A
Hollenbeck et al. (2014)	Observational	Early/late or no CAG	Immediately or during hypothermia, ≥24 h	122/144	61/63	68/73	54/59	100/100	33/39	≥3
Kern, et al. (2015)	Observational	Early/late or no CAG	<2 h, not specified	183/364	N/A	N/A	N/A	N/A	N/A	>3
Kleissner, et al. (2015)	Observational	Early/late or No CAG	<2 h, not specified	25/74	59/58	92/74	52/42	88/69	N/A	N/A
Reynolds et al. (2014)	Observational	Early/late or no CAG	Occurring directly from the Emergency Department, ICU, or referring facility, not specified	128/63	63/60	N/A	N/A	N/A	N/A	N/A
Song (2021)	Observational	Early/late or no CAG	≤24 h, >24 h	231/447	59/60	78/71	67/63	N/A	7/4	N/A
Kim (2019)	Observational	Early/late or no CAG	<2 h, 2–24 h	112/115	57/57	76/74	51/50	N/A	25/18	N/A
Patterson et al. (2017) ARREST	RCT multicentre	Early/late or no CAG	Not available	18/18	N/A	N/A	N/A	N/A	39/33	N/A
Hauw-Berlemont et al. (2022) EMERGE	RCT multicentre	Early/late or no CAG	Not specified, 48–96 h	141/138	65/64	73/67	75/80	7/4	30/23	≥3
Elfwen et al. (2019) DISCO	RCT multicentre	Early/late or no CAG	Immediately, ≥3 days	38/40	71/70	58/77	74/75	53/55	10/17	>3
Kern et al. (2020) PEARL	RCT multicentre	Early/late or no CAG	<2 h, ≥6 h	49/50	65/66	86/72	75/67	69/82	N/A	N/A
Lemkes et al. (2019) COACT	RCT multicentre	Early/late or no CAG	<2 h, after discharge from ICU	273/265	66/65	82/76	N/A	N/A	17/23	≥3
Desch et al. (2021) TOMAHA WK	RCT multicentre	Early/late or no CAG	Not specified	265/265	69/71	70/70	57/60	N/A	18/16	≥3
VianaTejedor et al. (2022) COUPE	RCT multicentre	Early/late or no CAG	<2 h, after neurological recovery	34/32	71/69	N/A	N/A	N/A	N/A	N/A

### Quality analysis

The risk of bias in RCTs was assessed using the Cochrane Risk of Bias tool (Supplementary Figure 1) while the Newcastle-Ottawa scale was utilized to assess the quality of observational studies included in our meta-analysis (Supplementary Table 2). Additionally, funnel plots were used to analyse publication bias for all outcomes where findings from ten or more studies could be meta-analysed.

### Statistical analysis

Review Manager (version 5.3; Copenhagen: The Nordic Cochrane Centre, The Cochrane Collaboration, 2014) was used to analyse most of the data included in our analysis. The random effects model was used for data analysis and dichotomous outcomes were pooled using the Mantel-Haenszel method. Odds ratios and standardized mean differences, together with their 95% confidence intervals (95% CIs), were used to report pooled results of dichotomous and continuous outcomes, respectively. A *p* value < 0.05 was considered significant. Heterogeneity was evaluated using the Higgins *I*^2^ statistic with *I*^2^ > 50% being considered significant heterogeneity. A sensitivity analysis was performed by removing one study at a time to assess whether the pooled result was inordinately impacted by any one particular study. Furthermore, the OpenMeta analyst software was used to perform meta-regression with variables including baseline hypertension, T2DM, history of previous stroke, and follow-up intervals. Forest plots and bubble scatter plots were used to visually assess the results of meta-analysis and meta-regression, respectively.

## Results

### Literature search

**Figure 1:**
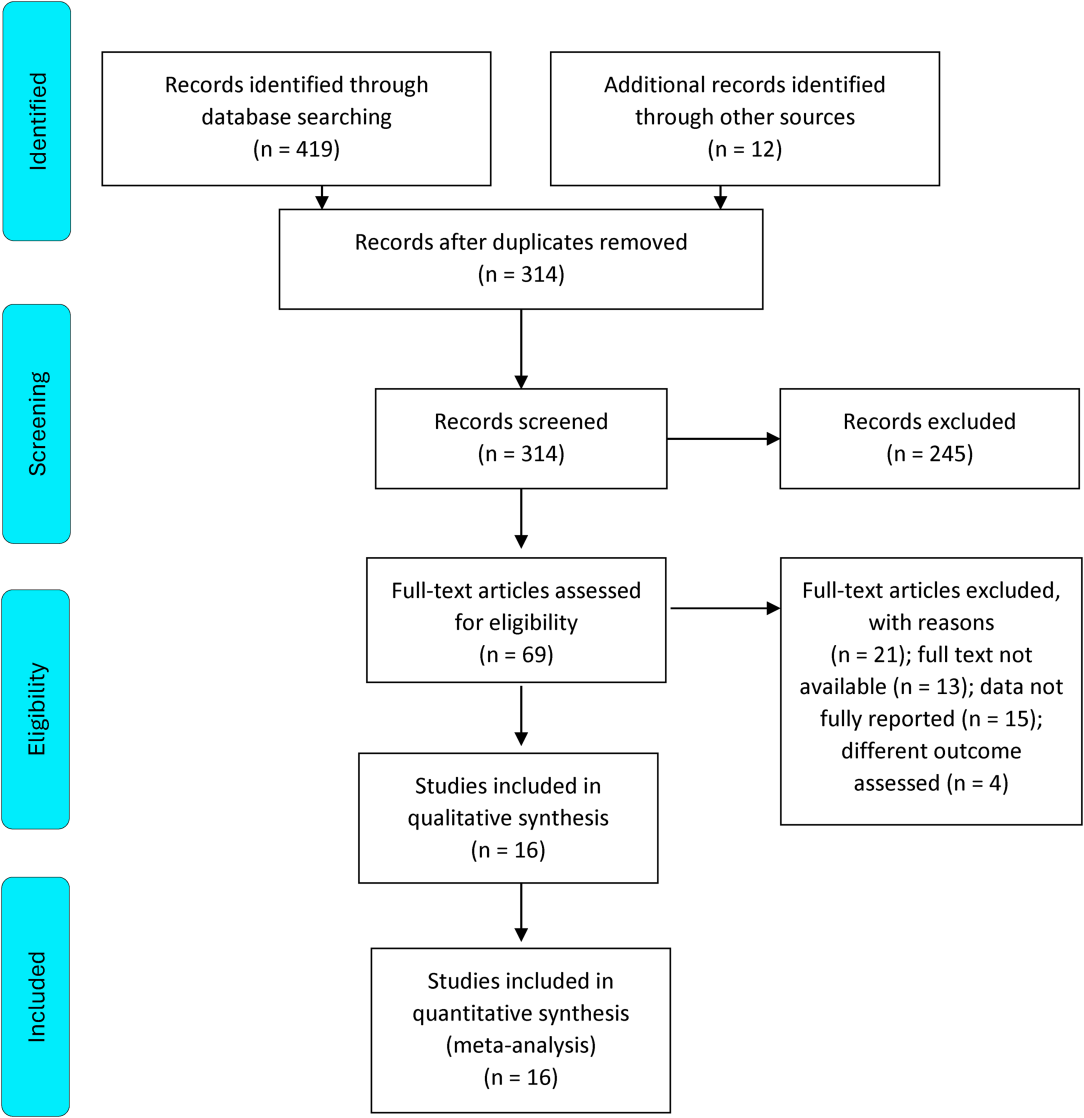
PRISMA 2020 flow diagram for new systematic reviews which included searches of databases and registers only.

The initial literature search yielded 431 potentially relevant articles. After application of the predetermined eligibility criterion, 16 studies (7 RCTs and 9 observational studies) were included in our meta-analysis (10–25). The PRISMA flowchart (Figure 1) details the screening and study selection process.

### Study characteristics and quality assessment

#### Effect on short-term mortality

**Figure 2:**
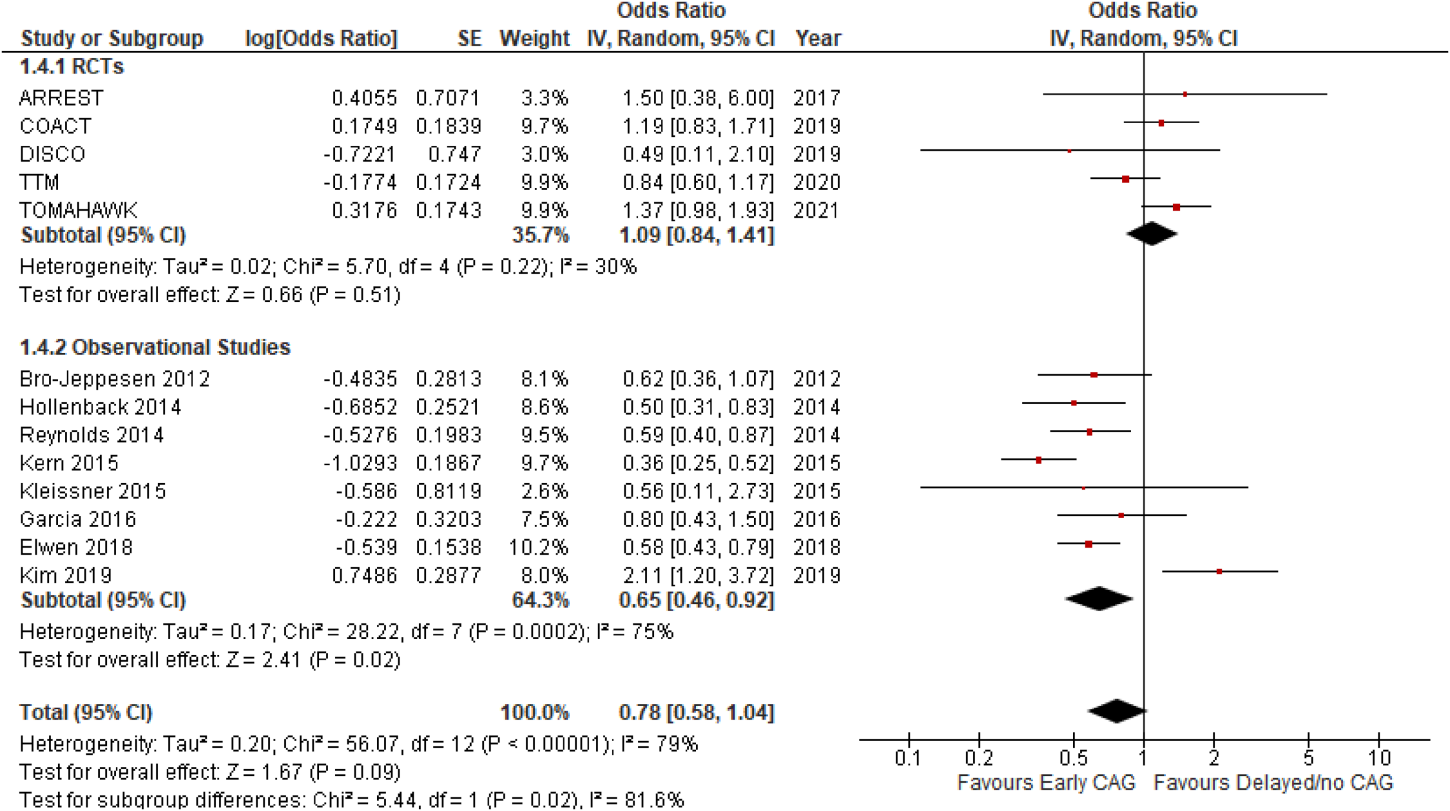
Forest plot comparing the effect of early CAG and delayed/no CAG on short term mortality among NSTE-OHCA patients in RCTs and observational studies.

**Figure 3:**
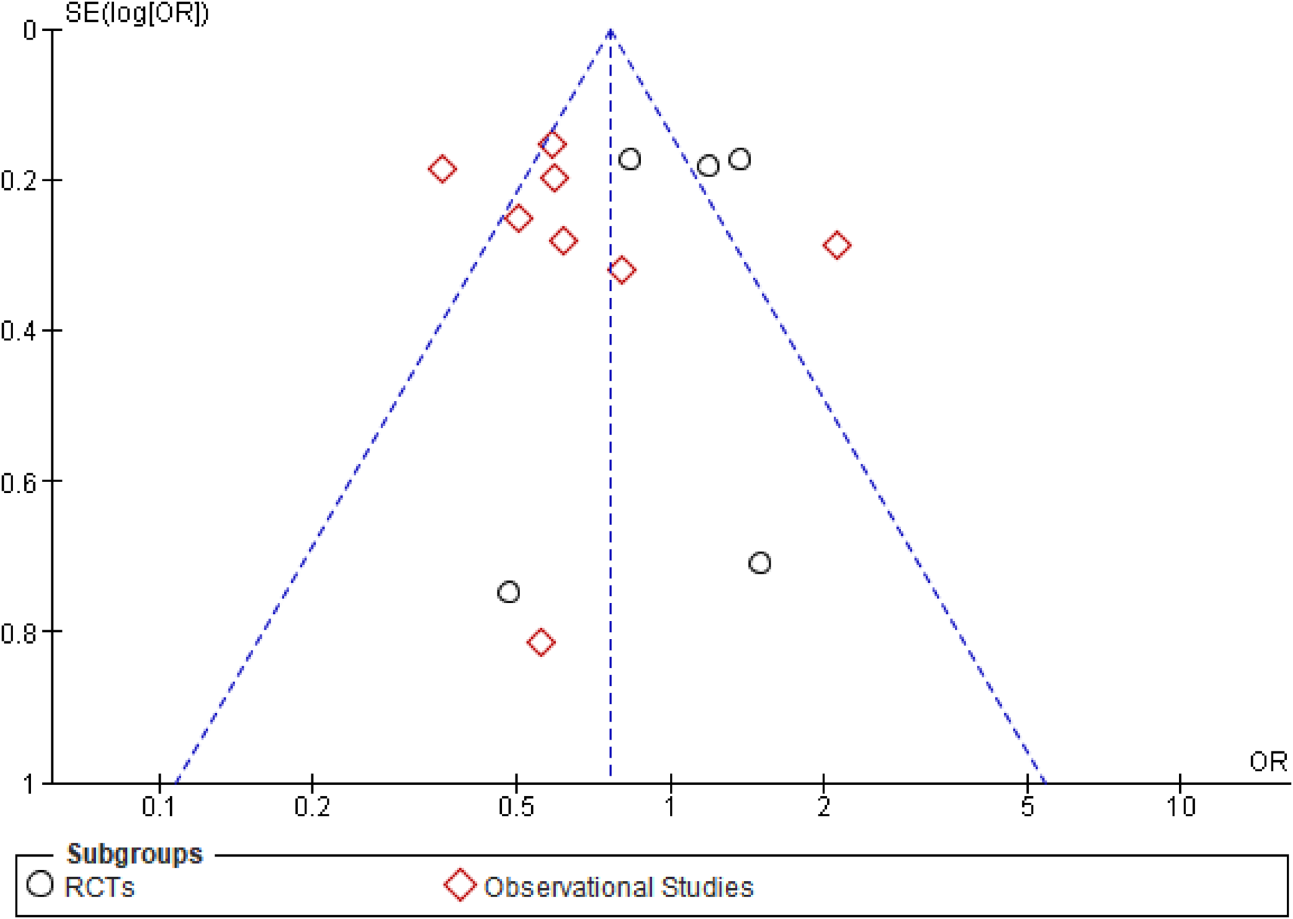
Funnel plot demonstrating publication bias for the outcome of short-term mortality.

This outcome was reported by 13 studies inclusive of 5 RCTs and 8 observational studies (Figure 2). The overall effect of early CAG in reducing the risk of short-term mortality was similar to that of delayed CAG (OR: 0.78; 95% CI: 0.58–1.04; *p* = 0.09; *I*^2^ = 79%) both overall as well as in the RCTs subgroup (OR: 1.09; 95% CI: 0.84–1.41; *p* = 0.51; *I*^2^ = 30%). However, among observational studies, early CAG significantly reduced the risk of short-term mortality when compared to delayed CAG (OR: 0.65; 95% CI: 0.46–0.92; *P* = 0.02; *I*^2^ = 79%). Additionally, significant subgroup differences (*p* = 0.02; *I*^2^ = 81.6%) were observed, and on performing a sensitivity analysis for observational studies, removing the study by Kim et al. reduced the heterogeneity (OR: 0.53; 95% CI: 0.44–0.65; *P* < 0.00001; *I*^2^ = 16%). Meta-regression analyses revealed T2DM to be a significant positive predictor of short-term mortality (9 studies; *p* = 0.006) (Supplementary Figure 2), while the funnel plot demonstrated no publication bias (Figure 3).

#### Effect on long-term mortality

**Figure 4:**
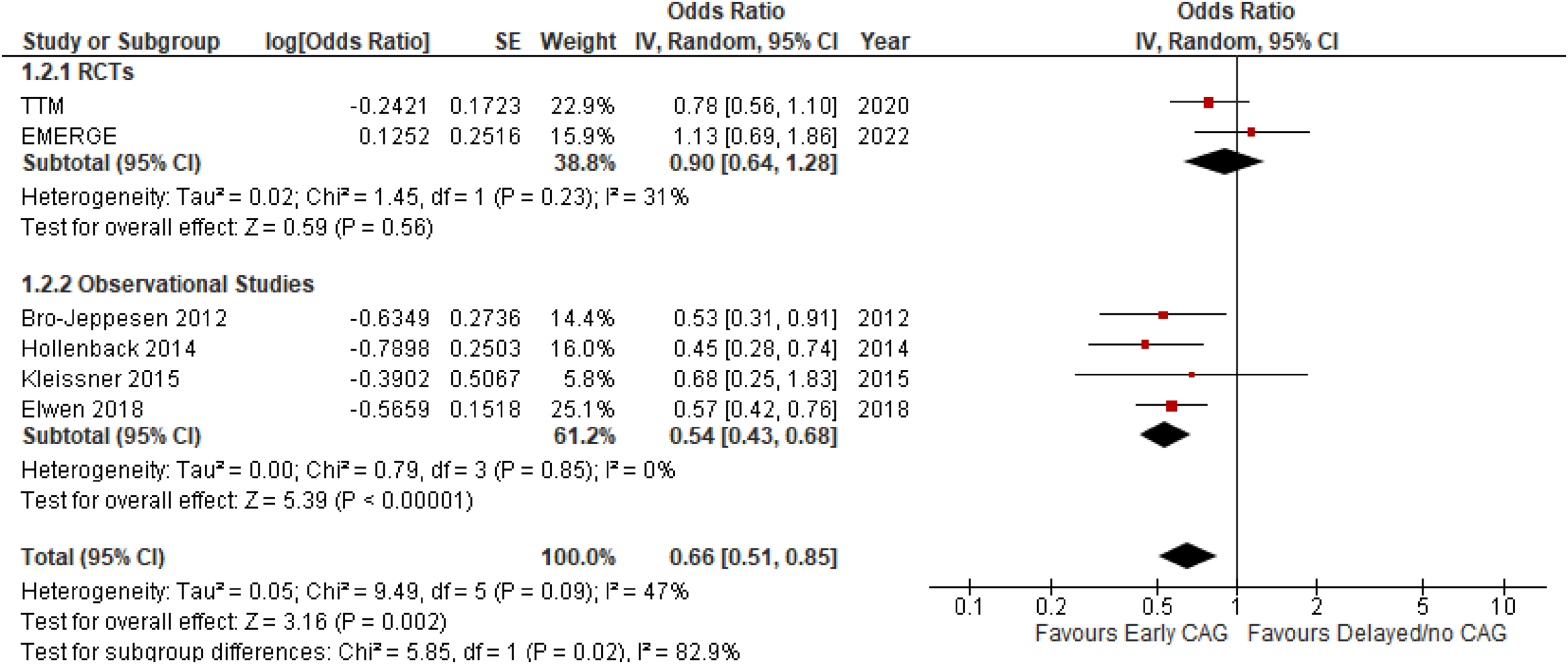
Forest plot comparing the effect of early CAG and delayed/no CAG on long term mortality among NSTE-OHCA patients in RCTs and observational studies. TTM, target temperature management after cardiac arrest; EME.GE, emergency versus delayed coronary angiogram in survivors of out-of-hospital cardiac arrest.

This outcome was reported by 6 studies inclusive of 2 RCTs and 4 observational studies (Figure 4). Early CAG significantly reduced the risk of long-term mortality, as compared to delayed CAG (OR: 0.66; 95% CI: 0.51–0.85; *p* = 0.002; *I*^2^ = 47%), both overall and in the observational studies’ subgroup (OR: 0.54; 95% CI: 0.43–0.68; *P* < 0.00001; *I*^2^ = 0%). However, significant subgroup differences were observed (*p* = 0.02; *I*^2^ = 82.9%) and in the RCTs’ subgroup, the effect of early and delayed CAG in reducing the risk of long-term mortality (OR: 0.90; 95% CI: 0.64–1.28; *p* = 0.56; *I*^2^ = 31%) remained similar.

#### Occurrence of a CPC 1–2 score at discharge

**Figure 5:**
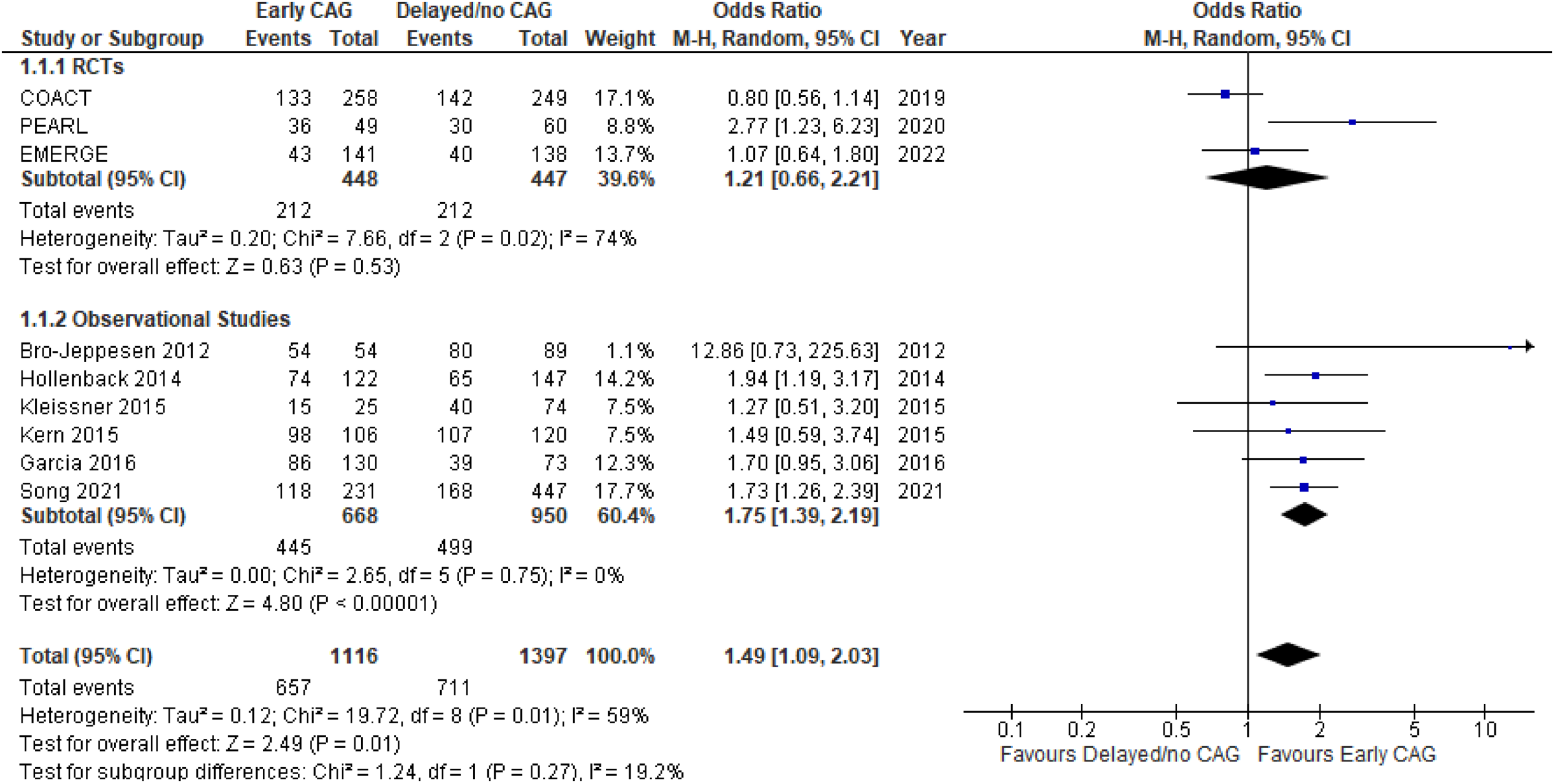
Forest plot comparing the effect of early CAG and delayed/no CAG on the incidence of CPC 1–2 scores at discharge among NSTE-OHCA patients in RCTs and observational studies. COACT, coronary angiography after cardiac arrest; pearl, early coronary angiography versus delayed coronary angiography; EMERGE, EMERGEncy versus delayed coronary angiogram in survivors of out-of-hospital cardiac arrest.

This outcome was reported by 9 studies, inclusive of 3 RCTs and 6 observational studies (Figure 5). As compared to delayed/no CAG, early CAG significantly improved the likelihood of a CPC score 1–2, both overall (OR: 1.49; 95% CI: 1.09–2.03; *P* = 0.01; *I*^2^ = 59%) and in the observational studies’ subgroup (OR: 0.54; 95% CI: 0.43–0.68; *P* < 0.00001; *I*^2^ = 0%). In the RCTs subgroup, however, the effect of early and delayed/no CAG was similar in improving the likelihood of a CPC score 1–2 (OR: 0.54; 95% CI: 0.43–0.68; *P* < 0.00001; *I*^2^ = 0%). No significant subgroup differences were noted (*p* = 0.27; *I*^2^ = 19.2%). Moreover, on performing sensitivity analysis, removing PEARL reduced the heterogeneity (OR: 0.88; 95% CI: 0.66–1.18; *P* = 0.39; *I*^2^ = 0%) in the RCTs subgroup, while results pertaining to observational studies remained unchanged. Meta-regression analyses revealed T2DM (5 studies; *p* < 0.001) **(**Supplementary Figure 3**)** and follow-up interval (6 studies; *p* = 0.004) to be significant positive and negative predictors of a CPC 1–2 score at discharge.

#### Occurrence of a CPC 1–2 score at follow-up

**Figure 6:**
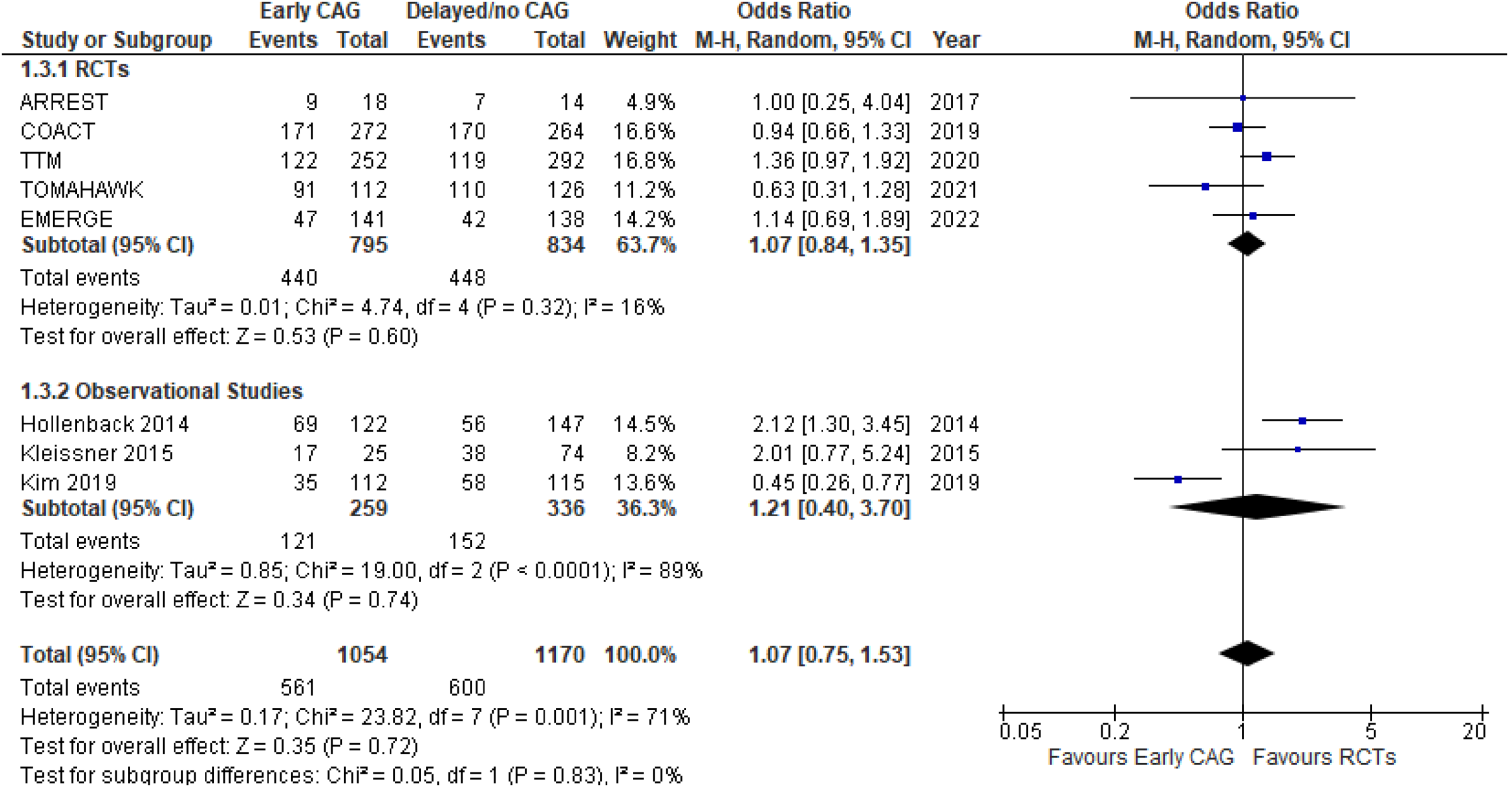
Forest plot comparing the effect of early CAG and delayed/no CAG on the incidence of CPC 1–2 scores at follow-up among NSTE-OHCA patients in RCTs and observational studies. ARREST, advanced reperfusion strategies for refractory cardiac arrest; COACT, coronary angiography after cardiac arrest; TTM, target temperature management after cardiac arrest; TOMAHAWK, immediate unselected coronary angiography versus delayed triage in survivors of out-of-hospital cardiac arrest without ST-segment elevation; EMERGE, EMERGEncy versus delayed coronary angiogram in survivors of out-of-hospital cardiac arrest.

This outcome was reported by 8 studies, inclusive of 5 RCTs and 3 observational studies (Figure 6). The effect of early CAG in improving the likelihood of CPC scores 1–2 was similar to delayed/no CAG, both overall (OR: 1.07; 95% CI: 0.75–1.53; *P* = 0.72; *I*^2^ = 71%), as well as in the individual RCTs’ (OR: 1.07; 95% CI: 0.84–1.35; *P* = 0.60; *I*^2^ = 16%) and observational studies’ (OR: 1.21; 95% CI: 0.40–3.70; *P* = 0.74; *I*^2^ = 89%) subgroups. There were no significant subgroup differences (*p* = 0.83; *I*^2^ = 0%) observed, and on conducting a sensitivity analysis for the observational studies, removing the study by Kim et al. decreased the heterogeneity (OR: 2.09; 95% CI: 1.35–3.24; *P* = 0.0009; *I*^2^ = 0%). Meta-regression analyses revealed T2DM and follow-up time to be a significant negative predictor of a CPC 1–2 score at follow-up (5 studies; *p* = 0.002) (Supplementary Figures 4, 5).

#### Occurrence of PCI after CAG

**Figure 7:**
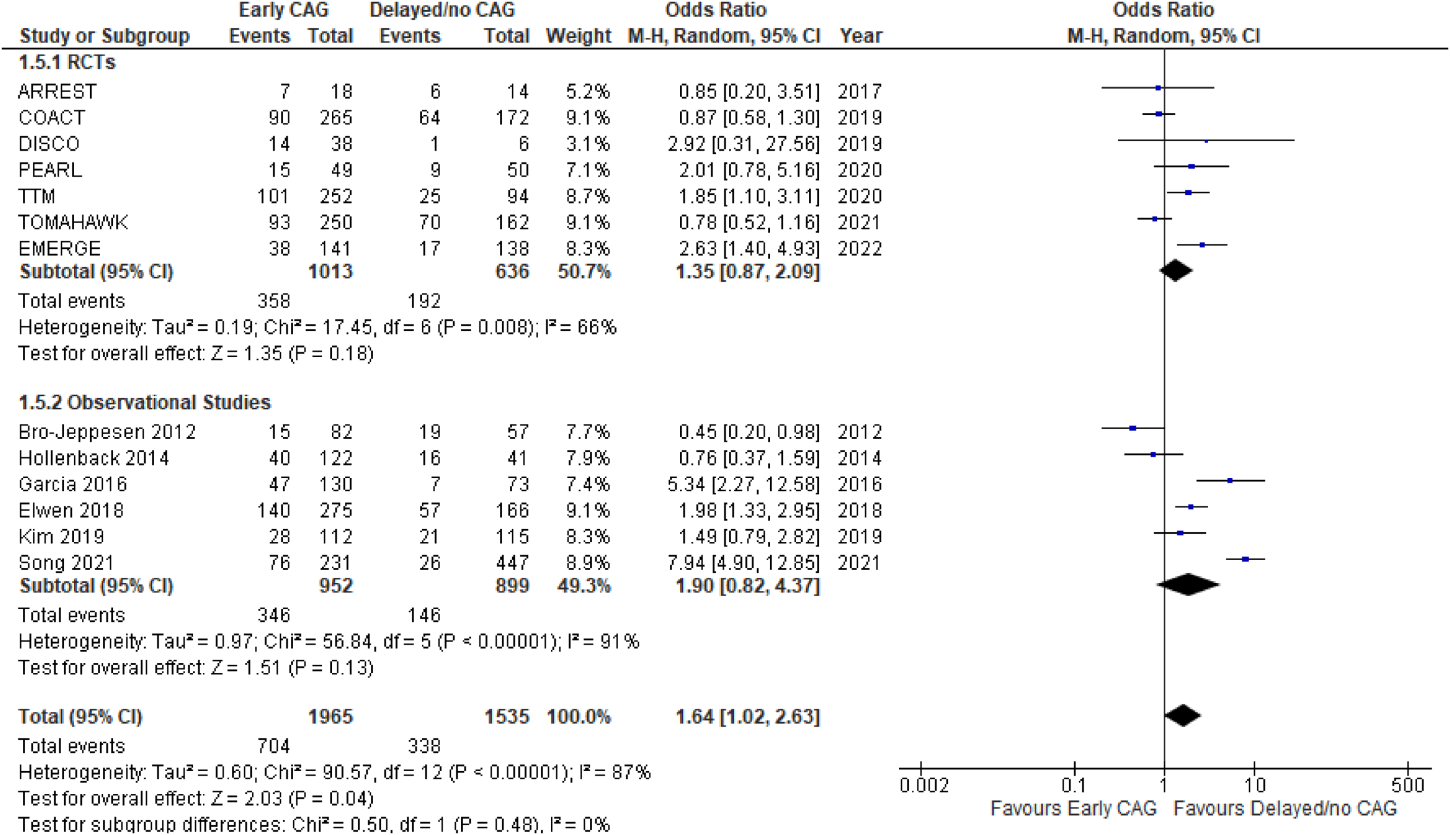
Forest plot comparing the effect on the occurrence of PCI after early CAG and delayed/no CAG among NSTE-OHCA patients in RCTs and observational studies. ARREST, advanced reperfusion strategies for refractory cardiac arrest; COACT, coronary angiography after cardiac arrest; DISCO, direct or subacute coronary angiography in out-of-hospital cardiac arrest; PEARL, early coronary angiography versus delayed coronary angiography; TTM, target temperature management after cardiac arrest; TOMAHAWK, immediate unselected coronary angiography versus delayed triage in survivors of out-of-hospital cardiac arrest without ST-segment elevation; EMERGE, EMERGEncy versus delayed coronary angiogram in survivors of out-of-hospital cardiac arrest.

**Figure 8:**
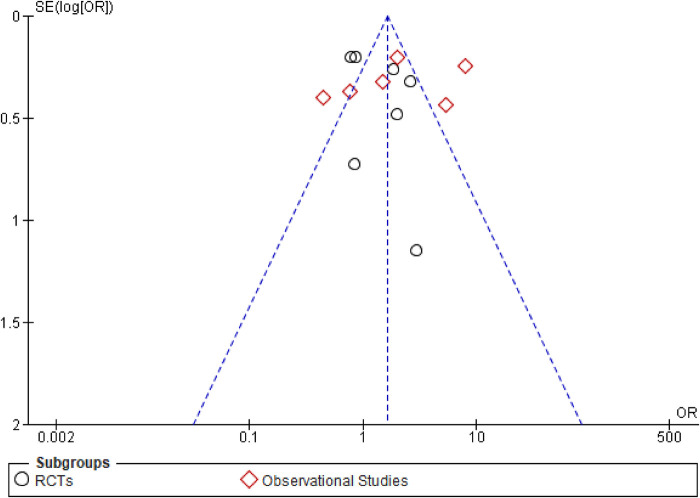
Funnel plot demonstrating publication bias for the outcome of successful PCI after CAG.

This outcome was reported by 13 studies, inclusive of 7 RCTs and 6 observational studies (Figure 7). Since PCI cannot be performed without a prior CAG, this outcome was strictly a comparison of patients undergoing early vs. delayed CAG only. Overall, delayed CAG significantly increased the incidence of a successful PCI as compared to those undergoing early CAG (OR: 1.64; 95% CI: 1.02–2.63; *P* = 0.04; *I*^2^ = 87%). However, this effect remained similar between early (OR: 1.21; 95% CI: 0.40–3.70; *P* = 0.74; *I*^2^ = 89%) and delayed CAG in the individual RCTs’ (OR: 1.35; 95% CI: 0.87–2.09; *P* = 0.18; *I*^2^ = 66%) and observational studies’ (OR: 1.90; 95% CI: 0.82–4.37; *P* = 0.13; *I*^2^ = 91%) subgroups. There were no significant subgroup differences (*p* = 0.48; *I*^2^ = 0%) and the funnel plot demonstrated publication bias (Figure 8).

## Discussion

In our comprehensive meta-analysis of 4,737 OHCA patients with NSTE, early CAG was associated with a significant reduction in the risk of short-term mortality, long-term mortality, and improved neurological outcome at discharge. Furthermore, the incidence of a successful PCI was significantly higher in patients who had delayed or no CAG compared to those who underwent early CAG. Notably, T2DM was a prominent confounder impacting results of short-term mortality, as well as CPC 1–2 score at discharge and follow-up.

According to ESC guidelines 2020, early coronary angiogram is not recommended in OHCA patients without STE due to mortality risk and deterioration in neurological function (26). These guidelines were based on the results of the COACT and TOMAHAWK trials, where early CAG was not significantly superior over delayed CAG (10, 13). In the COACT trial enrolling 552 NSTE-ACS patients, early CAG was not associated with improved 90-day survival benefit compared with delayed CAG (OR 0.89, 95% CI 0.621.27, *P* = 0.51). Similarly, in the TOMAHAWK trial, composite secondary endpoint of all-cause mortality or severe neurological deficit had significantly greater incidence in the early CAG group, indicating additional harm associated with immediate angiography.

In contrast, our current meta-analysis updated with the recent EMERGE trial showed no significant differences between both treatment strategies for short-term mortality in OHCA patients with NSTE. These findings are consistent with those of a previous meta-analysis by Verma et al., but in a larger pooled patient population (27). The results of our pooled analysis of observational studies additionally supports the use of early CAG in reducing risk of short and long-term mortality among OHCA patients. However, it is important to note that the long-term survival benefit of early CAG did not achieve statistical significance in the RCT subgroup.

According to meta-regression analysis, early CAG may likely not be associated with survival benefit at 30–90 days due to the presence of baseline covariates, like T2DM. Regression analysis demonstrated that among OHCA patients, those with T2DM had a significantly increasing risk of short-term mortality. Similar results were found in a meta-analysis evaluating the correlation between DM and poor outcomes in patients after OHCA and established a significant association (28). Pooled analysis of adjusted ORs demonstrated significantly reduced survival odds among DM patients after OHCA (OR 0.78, 95% CI, 0.68–0.89). In another observational study involving 28,955 OHCA patients, diabetes was associated with significantly decreased odds of 30-day survival (29). It is postulated that diabetic patients suffer from high rates of short-term mortality after OHCA due to metabolic derangement including increased blood glucose variability that can worsen prognosis (27). It is, therefore, crucial for clinicians to target appropriate management of diabetic patients to alleviate prognosis after OHCA, and concomitantly lower risk of short-term mortality after early CAG.

Early CAG was associated with an increased likelihood of a CPC 1–2 score at discharge in overall and observational studies’ subgroup. However, no significant differences in neurological outcomes were observed at follow-up. In contrast to our findings, a prior meta-analysis demonstrated no difference in neurological function between the two groups (27). Notably, previous meta-analyses have overlooked the assessment of CPC 1–2 scores at follow-up, which is crucial for a comprehensive evaluation of neurological functional recovery between patients undergoing early vs. delayed CAG (7, 27, 30).

It is worthwhile noting that in our study, T2DM was found to be a significant positive predictor of a CPC 1–2 score at discharge but a significant negative predictor of the same score on follow-up. Regardless, a potential explanation for such conflicting results may be the increased duration of T2DM at follow-up which may have led to microvascular pathologies that thereby deteriorated neurological function. This is in line with a recent study depicting a longer duration of DM to be associated with increased risks associated with OHCA (OR: 1.04, 95% CI: 1.02–1.06, per 1-year prevalence duration) (31). While our study demonstrated T2DM as being associated with better neurological outcomes at discharge, these results seem biologically implausible and in this light, it must be noted that our regression analysis was limited in that it was univariate. A multivariable meta-regression model may, in future, be a more reliable means of predicting the association between T2DM and a CPC 1–2 score while considering differences in patient characteristics at baseline between the two arms. Given this possibility as well as the well-established negative interaction between T2DM and cardiac disease (31, 32), the negative correlation of T2DM with long term neurological functional recovery should hold more weight and good diabetic control should be especially prioritized in OHCA patients. Nonetheless, it is crucial to interpret our findings here with caution as, being based off the regression of only five studies, they are statistically underpowered to be deemed reliably conclusive and accurate.

Since survivors of OHCA are usually comatose after resuscitation, PCI is the preferred revascularization technique. In our pooled analysis, we observed higher chances of a successful PCI in patients who underwent delayed or no CAG. These findings are inconsistent with those of a recent meta-analysis which demonstrated no difference in outcomes of coronary revascularisation (PCI or coronary artery bypass graft) in patients undergoing early vs. delayed CAG (RR 1.00; 95% CI 0.76–1.31; *I^2^* = 68%) (30). It is well-known that early CAG with PCI is beneficial only in the case where cardiac arrest is due to coronary artery disease. However, it is worth noting that in each of the COACT and TOMAHAWK trials, >50% patients did not have CAD. Thus, our findings should serve to clarify clinicians regarding the appropriate utilization of early CAG followed by PCI in NSTE OHCA.

Prior meta-analyses report conflicting results primarily due to the inclusion of patients with both STE and NSTE. Since both groups differ in terms of treatment, post-arrest clinical care and selection criteria, pooling them together can introduce confounding bias in results (33, 34). It is ideal to assess OHCA patients with NSTE as a separate group, as done in this study. Moreover, our meta-analysis is the first to incorporate results of the recently published DISCO trial (12), while additionally pooling results from observational studies to provide a holistic review of all data comparing early CAG to delayed CAG in OHCA patients with NSTE. We further observed a discrepancy in pooled analysis results between observational studies and RCTs. This may vastly be attributed to the differences in study design, and RCTs being underpowered, but also warrant future research to clarify whether the patient population in observational studies differs from RCTs, and if so, whether early CAG may be specifically beneficial in certain patient subgroups. Importantly, neurological function at follow-up was not significantly better in patients undergoing early CAG regardless of whether RCTs or observational studies were considered. This is in stark contrast to many previously published meta-analyses which analyse neurological function only at discharge or as a composite outcome (27, 30). Our study thereby negates the possibility of such a positive correlation, demanding more research to better explain neurological outcomes at different follow-up durations in our patient population. Additionally, however, the lack of individual patient-level data is an inherent limitation of our study, and there is a need for larger, more statistically powered trials and observational studies to definitively establish the comparison between outcomes of early vs. delayed CAG in NSTE OHCA patients.

## Conclusion

In conclusion, our results highlight the comparison between early and delayed/no CAG and in a nutshell, implicate the former to be better than the latter in terms of survival benefit. Furthermore, our findings indicate T2DM to be significantly predictive of short-term mortality and poor neurological functional recovery at follow-up after CAG. More research is however needed to elucidate specific high-risk patient subgroups and to corroborate our findings, specifically those pertaining to neurological functional recovery at discharge.

## Data Availability

The original contributions presented in the study are included in the article/Supplementary Material, further inquiries can be directed to the corresponding author.
