# Supplementary material for: Early coronary angiography in patients with myocardial infarction without ST elevation after out-of-hospital cardiac arrest: a systematic review and meta-analysis
Source: Front Cardiovasc Med. 2024 Nov 20;11:1374619. doi: 10.3389/fcvm.2024.1374619 (PMC11614880; doi:10.3389/fcvm.2024.1374619)

Supplementary Materials

Supplementary Table 1. Search Strategy Used in Each Database

Database (Articles Retrieved)	Search Strategy
MEDLINE (146 results)	("out-of-hospital cardiac arrest" OR "OHCA") AND ("non-ST segment elevation" OR "NSTE") AND ("early coronary angiography" OR "early CAG") AND ("patient outcomes" OR "mortality" OR "cerebral performance category" OR "percutaneous coronary intervention")
Cochrane Library (25 results)	("out-of-hospital cardiac arrest" OR "OHCA") AND ("non-ST segment elevation" OR "NSTE") AND ("early coronary angiography" OR "early CAG") AND ("patient outcomes" OR "mortality" OR "cerebral performance category" OR "percutaneous coronary intervention")

Supplementary Table 2. New Castle Ottawa Scale Assessment of Risk of Bias of Included Observational Studies

Study/Score	Selection				Comparability		Outcome			Total
	S1	S2	S3	S4	C1	C2	O1	O2	O3	
Bro Jeppensen et al.	*	*		*	*		*	*	*	7
Hollenback et al.	*				*	*	*	*	*	6
Garcia et al.	*	*	*		*	*	*	*	*	8
Elwen et al.	*				*	*	*	*	*	6
Kim et al.	*	*	*		*	*	*	*	*	8
Song et al.	*				*	*	*	*	*	6
Kern et al.	*	*		*	*		*	*	*	7
Kleisnner et al.	*				*	*	*	*	*	6
Reynolds et al.	*	*		*	*		*	*	*	7

Supplementary Figure 1. Assessment of Risk of Bias of Randomized Controlled Trials.

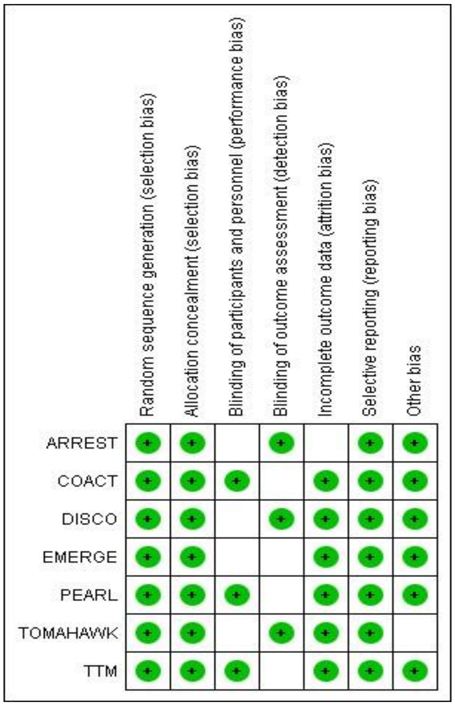

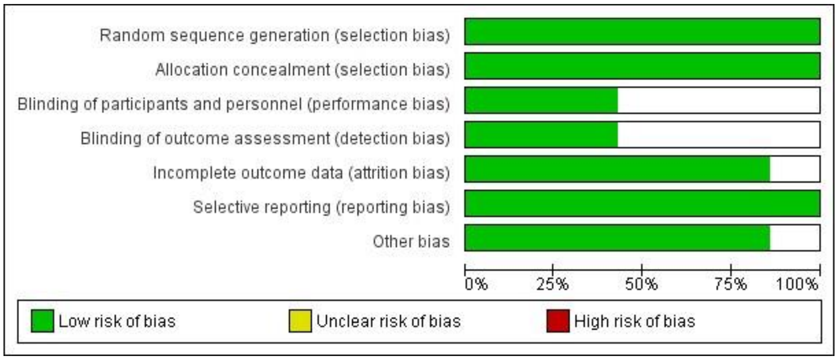

Supplementary Figure 2. Meta regression showing relationship between short term mortality and T2DM.

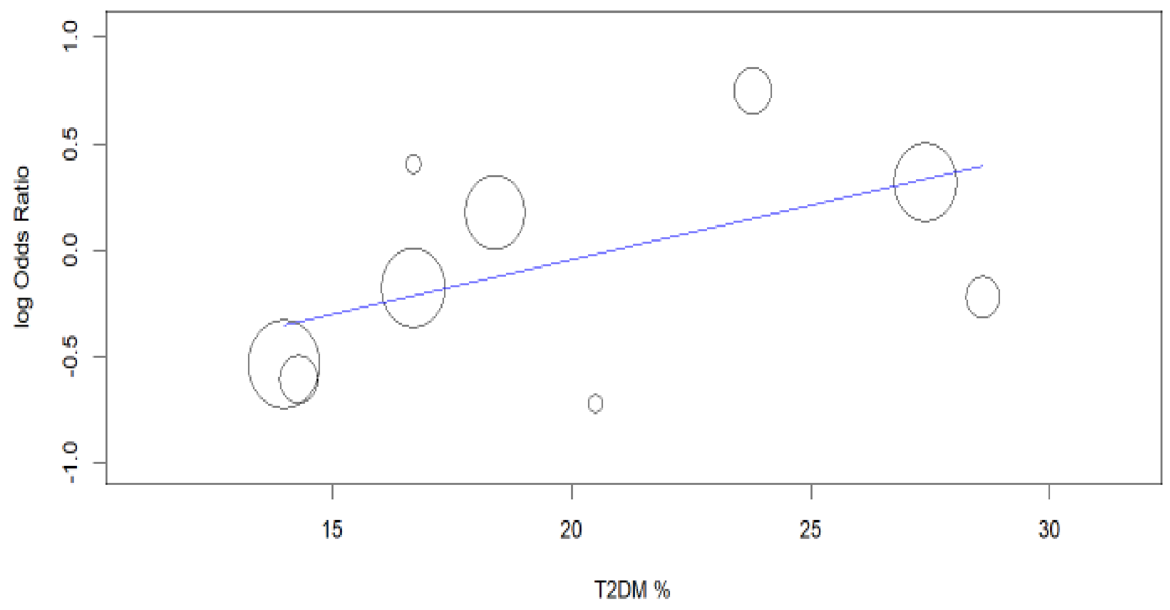

Supplementary Figure 3. Meta regression showing relationship between CPC 1-2 at discharge and T2DM.

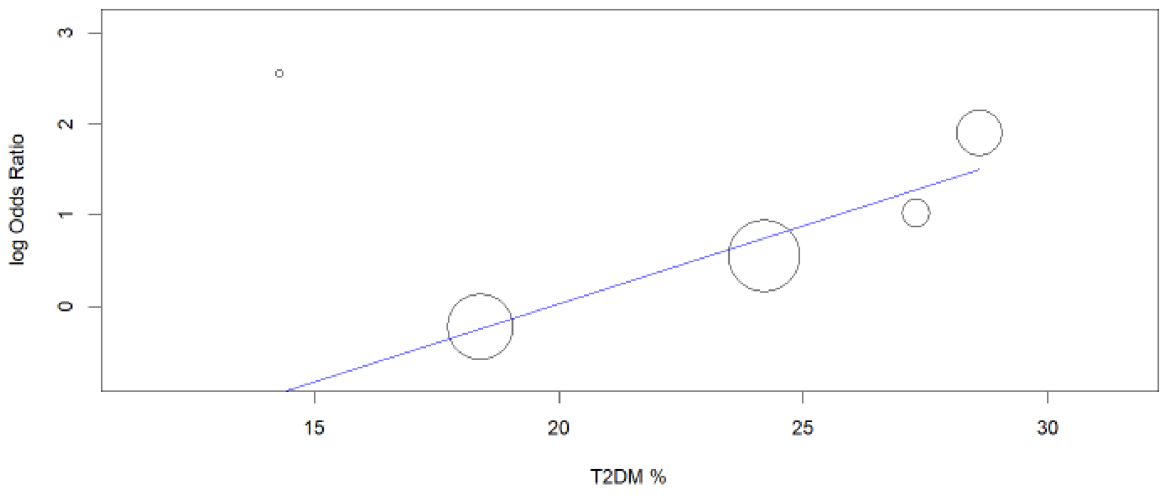

Supplementary Figure 4. Meta regression showing relationship between CPC 1-2 at F/U and T2DM.

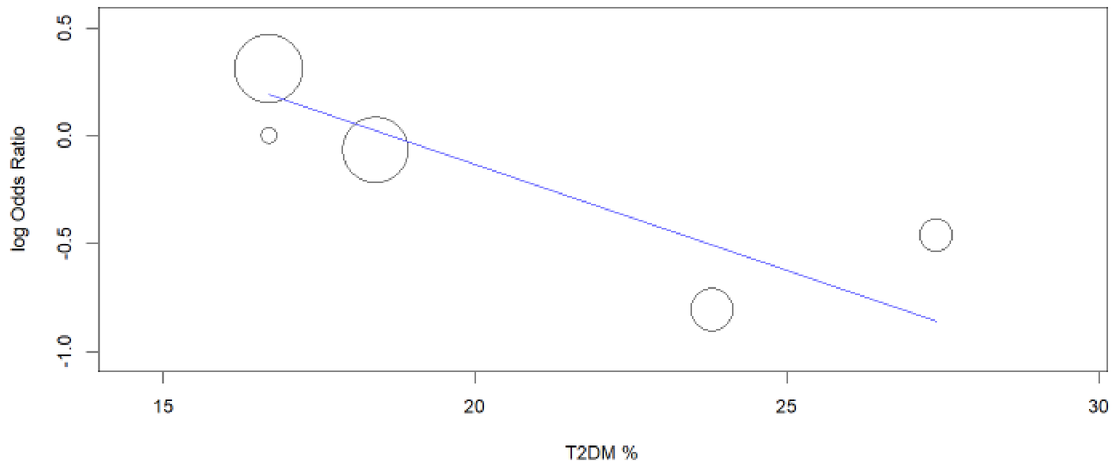

Supplementary Figure 5. Meta regression showing relationship between CPC 1-2 at discharge and follow-up time.

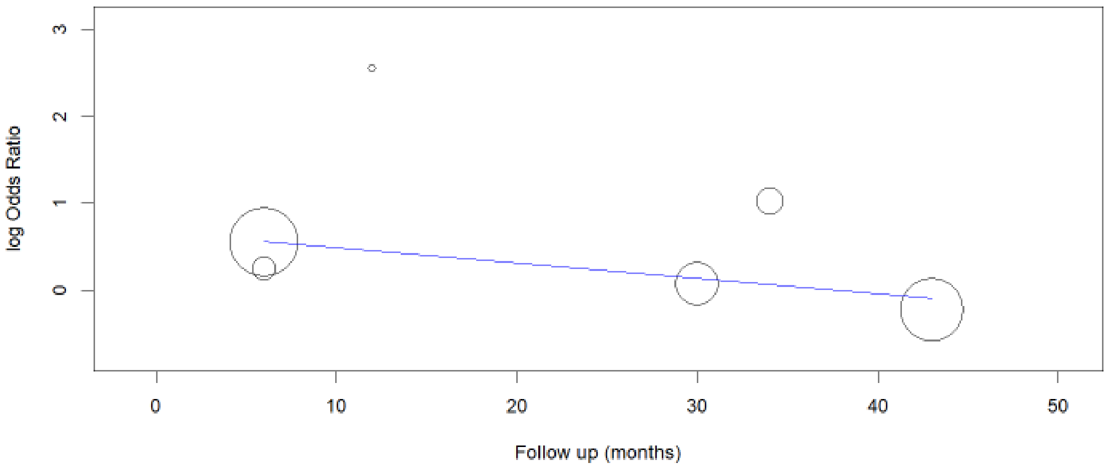

Supplement: Supplementary file 1 [file Datasheet1.pdf]
